# Supplementary material for: ZBP1 not RIPK1 mediates tumor necroptosis in breast cancer
Source: Nat Commun. 2021 May 11;12:2666. doi: 10.1038/s41467-021-23004-3 (PMC8113527; doi:10.1038/s41467-021-23004-3)
Supplement: Supplementary file 1 — Supplementary Information [file 41467_2021_23004_MOESM1_ESM.pdf]

Supplementary Information

Supplementary Fig. 1 RIPK1 is not involved in MVT-1 tumor necroptosis.

Supplementary Fig. 2 ZBP1 is highly increased in late stage of solid tumors.

Supplementary Fig. 3 ZBP1 is critical for tumor necroptosis.

Supplementary Fig. 4 Only glucose deprivation (GD) can induce necroptosis in primary mouse tumor cells.

Supplementary Fig. 5 Glucose deprivation (GD) promotes mitochondrial DNA release in primary mouse tumor cells.

Supplementary Fig. 6 Analysis of inflammatory cytokines in MVT-1 tumor model

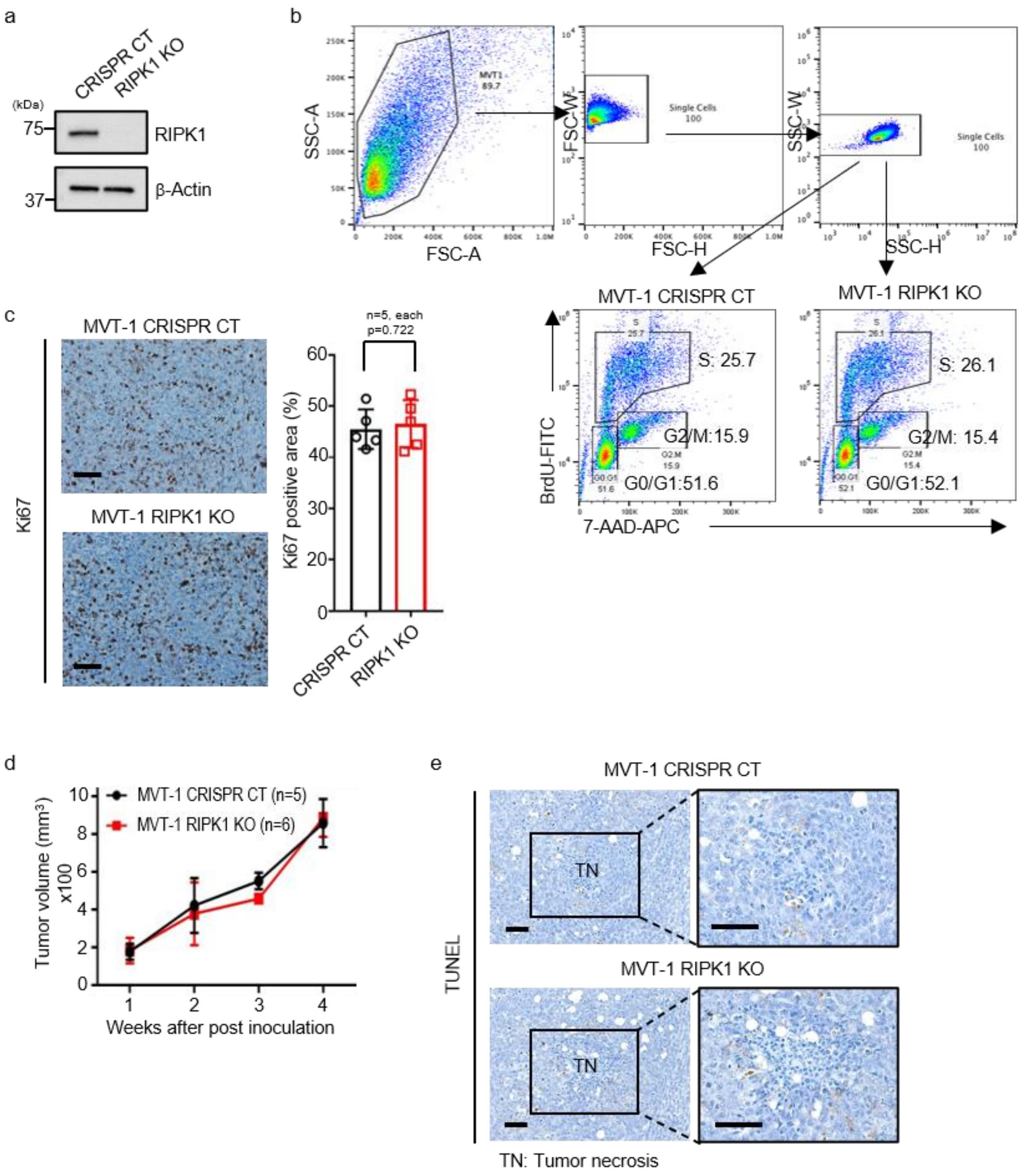

a
CRISPR CT
RIPK1 KO
(kDa)
75
RIPK1
37
β-Actin
b
SSC-A
FSC-A
MVT1
89.7
FSC-W
FSC-H
Single Cells
100
SSC-W
SSC-H
Single Cells
100
MVT-1 CRISPR CT
MVT-1 RIPK1 KO
S: 25.7
G2/M:15.9
G0/G1:51.6
S: 26.1
G2/M: 15.4
G0/G1:52.1
BrdU-FITC
7-AAD-APC
c
MVT-1 CRISPR CT
MVT-1 RIPK1 KO
Ki67
n=5, each
p=0.722
Ki67 positive area (%)
CRISPR CT
RIPK1 KO
d
MVT-1 CRISPR CT (n=5)
MVT-1 RIPK1 KO (n=6)
Tumor volume (mm³) x100
Weeks after post inoculation
e
MVT-1 CRISPR CT
MVT-1 RIPK1 KO
TUNEL
TN
TN: Tumor necrosis

Supplementary Fig. 1 RIPK1 is not involved in MVT-1 tumor necroptosis. a, MVT-1 CRISPR/Cas9 control (CRISPR CT) or MVT-1 RIPK1 knock out (RIPK1 KO) cells were generated by using CRISPR/Cas9 system and then examined by Western blot analysis using the indicated antibodies. Western blotting analysis is representative of three independent experiments. b, FACS sequential gating/sorting strategies are presented. Proliferation and cell cycle of MVT-1 CRISPR CT or MVT-1 RIPK1 KO cells were analyzed by BrdU-FITC uptake and 7-AAD-APC staining using flow cytometry. c, Representative images of Ki67 stained 4-week tumor sections from mice implanted with MVT-1 CRISPR CT or MVT-1 RIPK1 KO cells (left panel). Scale bar, 50 µm. Ki67 positive area was quantified using Image J program (right panel). Data are presented as mean values +/- SEM. d, Tumor volume in FVB/NJ mice implanted with MVT-1 CRISPR CT or MVT-1 RIPK1 KO cells was measured weekly until 4 weeks. Data are presented as mean values +/- SEM. e, Representative image of 4-week tumor sections identifying areas of apoptosis by TUNEL staining from mice implanted as in Supplementary Fig. 1c. Scale bar, 50 µm. The data results representative of three independent experiments. Two-sided student's t-test was used to determine the statistical significance of differences between groups. Differences with P values < 0.05 were considered significant. Source data are provided as a Source Data file.

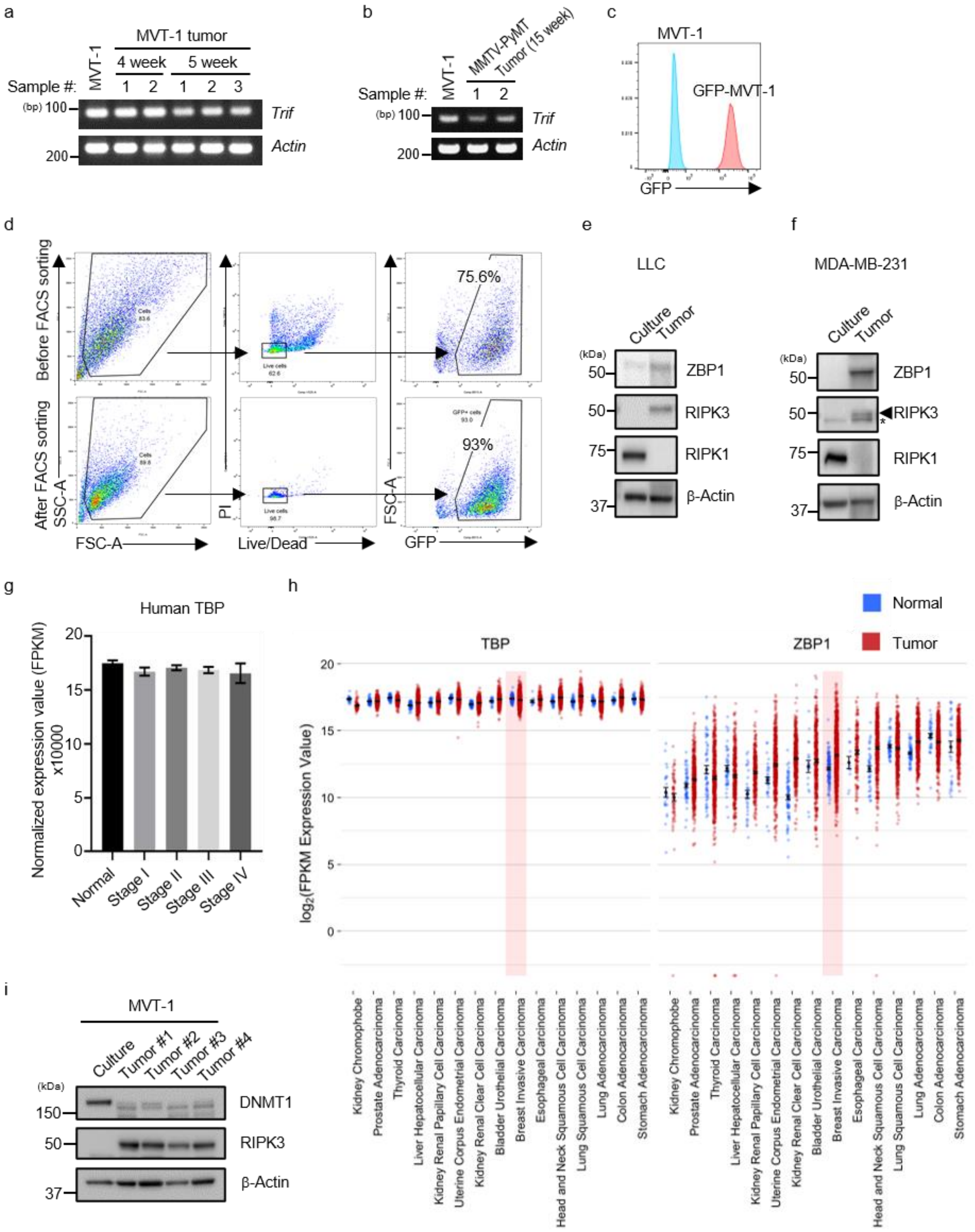

a
MVT-1 tumor
4 week
5 week
Sample #:
MVT-1
1 2 1 2 3
(bp) 100
200
Trif
Actin
b
MMTV-PyMT Tumor (15 week)
Sample #:
MVT-1
1 2
(bp) 100
200
Trif
Actin
c
MVT-1
GFP-MVT-1
GFP
d
Before FACS sorting
After FACS sorting
75.6%
93%
SSC-A
FSC-A
PI
Live/Dead
FSC-A
GFP
e
LLC
Culture
Tumor
(kDa)
50
50
75
37
ZBP1
RIPK3
RIPK1
β-Actin
f
MDA-MB-231
Culture
Tumor
(kDa)
50
50
75
37
ZBP1
RIPK3
*
RIPK1
β-Actin
g
Human TBP
Normalized expression value (FPKM) x10000
20
15
10
5
0
Normal
Stage I
Stage II
Stage III
Stage IV
h
Normal
Tumor
TBP
ZBP1
log2(FPKM Expression Value)
20
15
10
5
0
Kidney Chromophobe
Prostate Adenocarcinoma
Thyroid Carcinoma
Liver Hepatocellular Carcinoma
Kidney Renal Papillary Cell Carcinoma
Uterine Corpus Endometrial Carcinoma
Kidney Renal Clear Cell Carcinoma
Bladder Urothelial Carcinoma
Breast Invasive Carcinoma
Esophageal Carcinoma
Head and Neck Squamous Cell Carcinoma
Lung Squamous Cell Carcinoma
Lung Adenocarcinoma
Colon Adenocarcinoma
Stomach Adenocarcinoma
i
MVT-1
Culture
Tumor #1
Tumor #2
Tumor #3
Tumor #4
(kDa)
150
50
37
DNMT1
RIPK3
β-Actin

Supplementary Fig. 2 ZBP1 is highly increased in late stage of solid tumors. a-b, The PCR results representative of three independent experiments. a, *Trif* or *Actin* mRNA expression level of MVT-1 tumor sample collected at 4 and 5 weeks from mice implanted with MVT-1 cells were detected by reverse transcription PCR. b, *Trif* or *Actin* mRNA expression level of MMTV-PyMT tumor sample collected at 15 weeks mice were detected by reverse transcription PCR. c, FACS analysis of GFP vector transfected MVT-1 tumor cells. d, FACS sequential gating/sorting strategies are presented. GFP tagged MVT-1 tumor cells from 5 weeks tumors were collected and sorted from total tumor cells using a BD FACSAria Fusion. e, Western blotting analysis representative of three independent experiments of LLC cell lysates and LLC tumor cell lysates. The lysates were collected from 4-week tumors of C57BL/6J mice implanted with LLC mouse lung cancer cells were done using the indicated antibodies. f, Western blotting analysis representative of three independent experiments of MDA-MB-231 cell lysates and MDA-MB-231 tumor cell lysates. The lysates were collected from 8-week tumors of BALB/c-nu/nu mice implanted with MDA-MB-231 human breast cancer cells were done using the indicated antibodies (*, non-specific band). To be comparable to the late stage necrotic tumors, the tumors from these models in e and f were collected when they approached 1500-2000 mm^3 in volume and had tumor necrosis. g, Differential expression of human TBP1 (control) was analyzed for human breast cancer dataset (TCGA-BRCA) and performed for each tumor stage separately (Normal stage, n=112; Stage I, n=182; Stage II, n=627; Stage III, n=249; Stage IV, n=20). Data are presented as mean values +/- SD. h, Selected TCGA datasets are shown along the x-axis with normal and tumor samples colored in blue and red, respectively. i, Western blotting analysis of MVT-1 cell lysates or MVT-1 tumor cell lysates collected from 5-week tumors of FVB/NJ mice implanted with MVT-1 cells using the indicated antibodies. Western blotting analysis representative of three independent experiments. Two-sided student's t-test was used to determine the statistical significance of differences between groups. Differences with P values < 0.05 were considered significant. Source data are provided as a Source Data file.

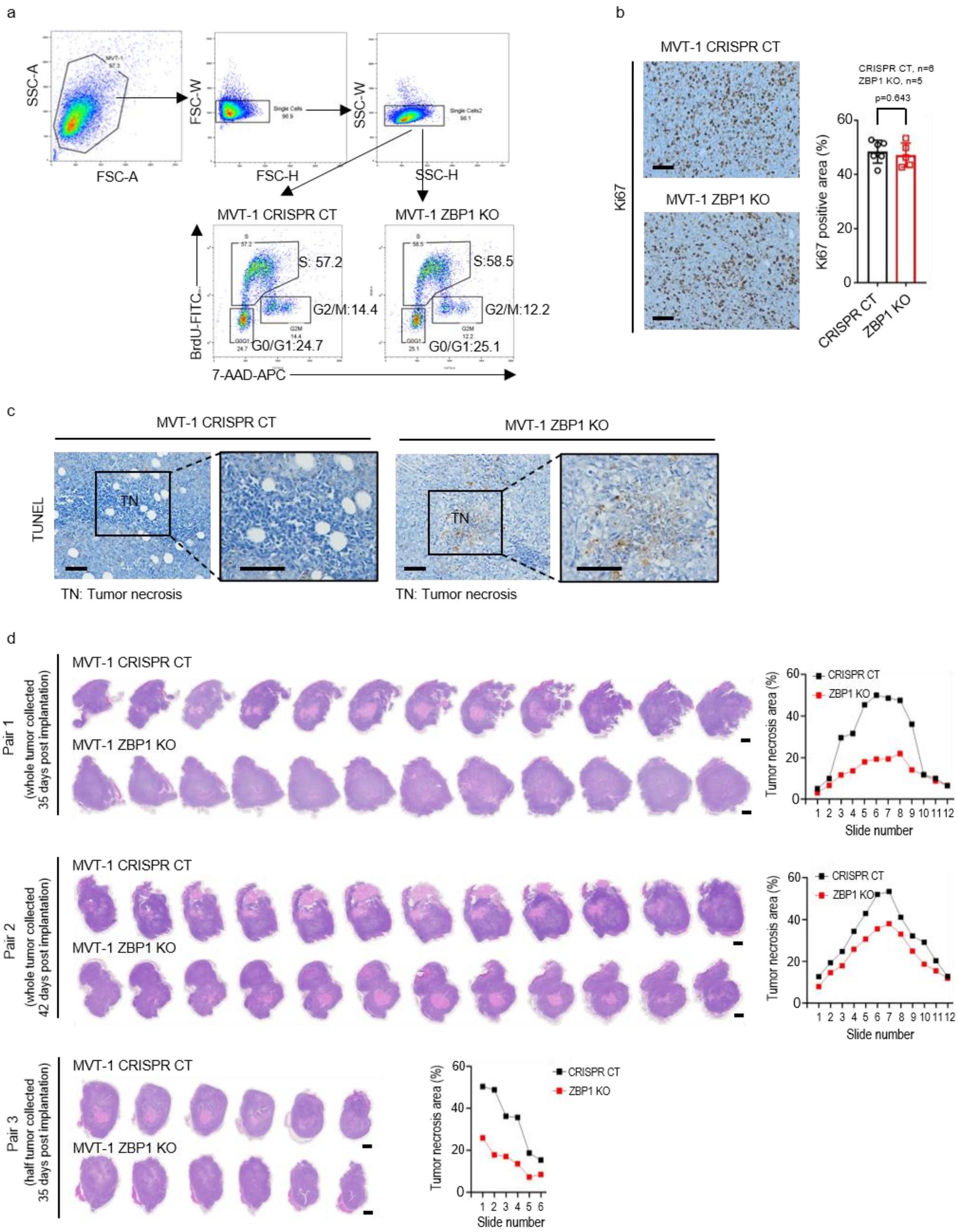

a
SSC-A
FSC-A
FSC-W
FSC-H
SSC-W
SSC-H
MVT-1 CRISPR CT
MVT-1 ZBP1 KO
BrdU-FITC
7-AAD-APC
S: 57.2
G2/M:14.4
G0/G1:24.7
S:58.5
G2/M:12.2
G0/G1:25.1
b
MVT-1 CRISPR CT
MVT-1 ZBP1 KO
Ki67
CRISPR CT, n=6
ZBP1 KO, n=5
p=0.643
Ki67 positive area (%)
60
40
20
0
CRISPR CT
ZBP1 KO
c
MVT-1 CRISPR CT
MVT-1 ZBP1 KO
TUNEL
TN
TN: Tumor necrosis
TN: Tumor necrosis
d
MVT-1 CRISPR CT
MVT-1 ZBP1 KO
Pair 1
(whole tumor collected 35 days post implantation)
Tumor necrosis area (%)
CRISPR CT
ZBP1 KO
Slide number
MVT-1 CRISPR CT
MVT-1 ZBP1 KO
Pair 2
(whole tumor collected 42 days post implantation)
Tumor necrosis area (%)
CRISPR CT
ZBP1 KO
Slide number
MVT-1 CRISPR CT
MVT-1 ZBP1 KO
Pair 3
(half tumor collected 35 days post implantation)
Tumor necrosis area (%)
CRISPR CT
ZBP1 KO
Slide number

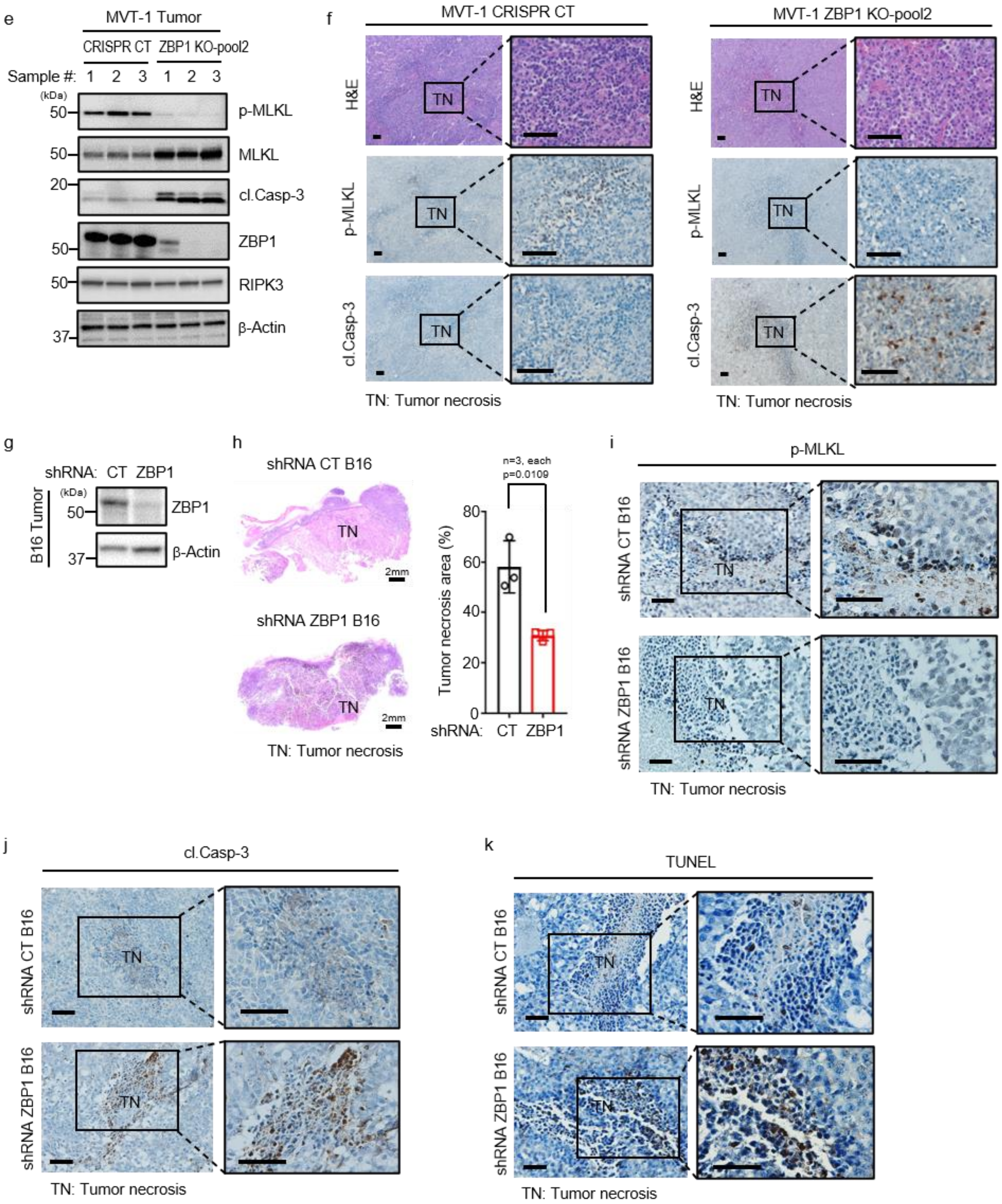

e
MVT-1 Tumor
CRISPR CT
ZBP1 KO-pool2
Sample #: 1 2 3 1 2 3
(kDa)
50
p-MLKL
MLKL
20
cl.Casp-3
ZBP1
RIPK3
37
β-Actin
f
MVT-1 CRISPR CT
MVT-1 ZBP1 KO-pool2
H&E
p-MLKL
cl.Casp-3
TN
TN: Tumor necrosis
g
shRNA: CT ZBP1
B16 Tumor
ZBP1
β-Actin
h
shRNA CT B16
shRNA ZBP1 B16
2mm
n=3, each
p=0.0109
Tumor necrosis area (%)
0 20 40 60 80
shRNA: CT ZBP1
i
p-MLKL
shRNA CT B16
shRNA ZBP1 B16
j
cl.Casp-3
k
TUNEL

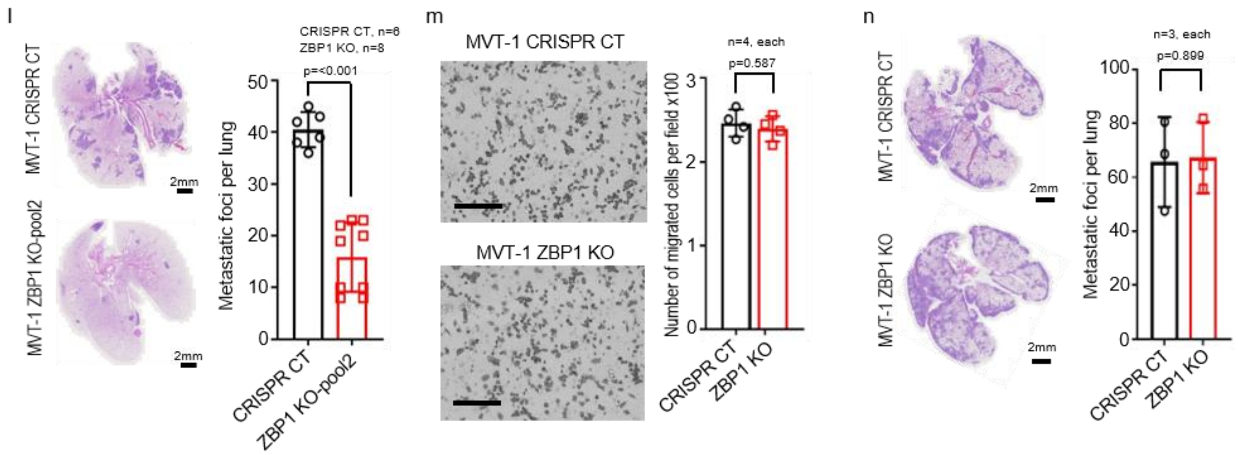

l
MVT-1 CRISPR CT
MVT-1 ZBP1 KO-pool2
2mm
2mm
CRISPR CT, n=6
ZBP1 KO, n=8
p=<0.001
Metastatic foci per lung
CRISPR CT
ZBP1 KO-pool2
m
MVT-1 CRISPR CT
MVT-1 ZBP1 KO
n=4, each
p=0.587
Number of migrated cells per field x100
CRISPR CT
ZBP1 KO
n
MVT-1 CRISPR CT
MVT-1 ZBP1 KO
2mm
2mm
n=3, each
p=0.899
Metastatic foci per lung
CRISPR CT
ZBP1 KO

Supplementary Fig. 3 ZBP1 is critical for tumor necroptosis. a, FACS sequential gating/sorting strategies are presented. Proliferation and cell cycle of MVT-1 CRISPR CT or MVT-1 ZBP1 KO cells was analyzed by BrdU-FITC uptake and 7-AAD-APC staining by flow cytometry. b, Representative images from three independent experiments of Ki67 stained 5-week tumor sections from FVB/NJ mice implanted with MVT-1 CRISPR CT or MVT-1 ZBP1 KO cells (left panel). Scale bar, 50 µm. Ki67 positive area was quantified using Image J program (right panel). Data are presented as mean values +/- SEM. c, Representative images from three independent experiments of tumor sections identifying areas of apoptosis by TUNEL staining from mice implanted as in Supplementary Fig. 3b. Scale bar, 50 µm. d, Representative images from three independent experiments of whole tumor sections (500 µm apart with 6-12 levels) stained with H&E from mice implanted with CRISPR CT or ZBP1 KO MVT-1 cells. Pair 1 and 3 tumors were collected at 35 days after implanted with CRISPR CT or ZBP1 KO. Pair 2 tumors were collected at 42 days after implanted with CRISPR CT or ZBP1 KO. Pair 3 images represent sections from one half of the tumor collected. Right panel shows the percentage of tumor necrosis (TN) area of total tumor area in each section/slide. Scale bar, 2 mm e, Representative Western blotting analysis from three independent experiments of tumor lysates from tumors of FVB/NJ mice implanted with MVT-1 CRISPR CT or MVT-1 ZBP1 KO (ZBP1 KO-pool2 sequences are shown in Supplementary Table 1) cells, using the indicated antibodies. f, Representative images from three independent experiments of H&E and immunohistological stained tumor sections with phospho-MLKL (p-MLKL) or cleaved caspase-3 (cl.Casp-3) antibodies of 5-week tumor sections from mice implanted as in Supplementary Fig. 3e. Scale bar, 50 µm. g, B16 tumor lysates from C57BL/6J mice implanted with shRNA control or shRNA targeting ZBP1 were collected at 2-weeks post-implantation and examined by Western blotting analysis using the indicated antibodies. Western blotting analysis representative of three independent experiments. h, Representative images from three independent experiments of tumors stained with H&E from mice implanted as in Supplementary Fig. 3g (left panel, scale bar, 2 mm). Percentage of tumor necrosis (TN) area of total tumor area from mice at 2-week (right panel). Data are presented as mean values +/- SEM. i, Representative immunohistological images from three independent experiments of p-MLKL antibody stained tumor sections from mice implanted as in Supplementary Fig. 3g. Scale bar, 50 µm. j, Representative immunohistological images from three independent experiments cl.Casp-3 antibody stained 5-week tumor sections from mice implanted as in Supplementary Fig. 3g. Scale bar, 50 µm. k, Representative images of tumor sections identifying areas of apoptosis by TUNEL staining from mice implanted as in Supplementary Fig. 3g. Scale bar, 50 µm. l, Left panel shows the representative images from three independent experiments of H&E stained lung sections from mice implanted as in Supplementary Fig. 3e showing lung metastasis. Scale bar, 2 mm. Right panel shows the quantification of metastatic foci in lungs from mice at 5-week post-implantation. Data are presented as mean values +/- SEM. m, Representative image representative of three independent experiments of migrated MVT-1 CRISPR CT or MVT-1 ZBP1 KO cells analyzed by a transwell assay (left panel). Scale bar, 50 µm. Quantification of migrated cells per field

(right panel, n=4, each). Data are presented as mean values +/- SEM. n, Representative H&E images from three independent experiments of lungs collected from mice 2 weeks after being injected with 1×10^6 (*i.v.*), either MVT-1-CRISPR CT or MVT-1 ZBP1 KO cells (left panel). Scale bar, 2 mm. The number of metastatic foci per lung was quantified (right panel). Data are presented as mean values +/- SEM. Two-sided student's t-test was used to determine the statistical significance of differences between groups. Differences with P values < 0.05 were considered significant. Source data are provided as a Source Data file.

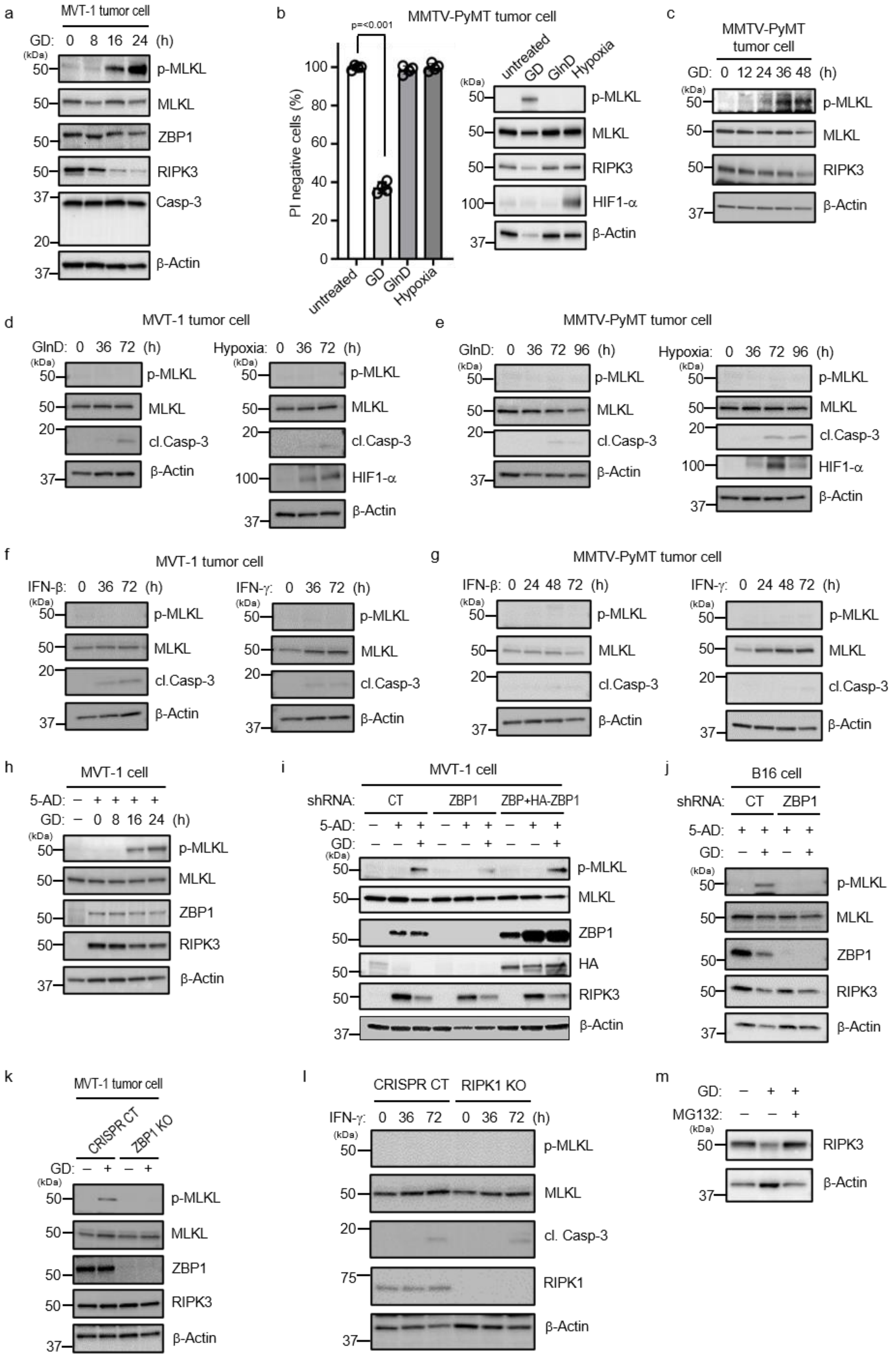
a
MVT-1 tumor cell
GD: 0 8 16 24 (h)
(kDa)
p-MLKL
MLKL
ZBP1
RIPK3
Casp-3
β-Actin
b
MMTV-PyMT tumor cell
p=<0.001
PI negative cells (%)
untreated
GD
GlnD
Hypoxia
p-MLKL
MLKL
RIPK3
HIF1-α
β-Actin
c
MMTV-PyMT tumor cell
GD: 0 12 24 36 48 (h)
p-MLKL
MLKL
RIPK3
β-Actin
d
MVT-1 tumor cell
GlnD: 0 36 72 (h)
Hypoxia: 0 36 72 (h)
p-MLKL
MLKL
cl.Casp-3
β-Actin
HIF1-α
e
MMTV-PyMT tumor cell
GlnD: 0 36 72 96 (h)
Hypoxia: 0 36 72 96 (h)
p-MLKL
MLKL
cl.Casp-3
β-Actin
HIF1-α
f
MVT-1 tumor cell
IFN-β: 0 36 72 (h)
IFN-γ: 0 36 72 (h)
p-MLKL
MLKL
cl.Casp-3
β-Actin
g
MMTV-PyMT tumor cell
IFN-β: 0 24 48 72 (h)
IFN-γ: 0 24 48 72 (h)
p-MLKL
MLKL
cl.Casp-3
β-Actin
h
MVT-1 cell
5-AD: – + + + +
GD: – 0 8 16 24 (h)
p-MLKL
MLKL
ZBP1
RIPK3
β-Actin
i
MVT-1 cell
shRNA: CT ZBP1 ZBP+HA-ZBP1
5-AD: – + + – + + – + +
GD: – – + – – + – – +
p-MLKL
MLKL
ZBP1
HA
RIPK3
β-Actin
j
B16 cell
shRNA: CT ZBP1
5-AD: + + + +
GD: – + – +
p-MLKL
MLKL
ZBP1
RIPK3
β-Actin
k
MVT-1 tumor cell
CRISPR CT
ZBP1 KO
GD: – + – +
p-MLKL
MLKL
ZBP1
RIPK3
β-Actin
l
CRISPR CT RIPK1 KO
IFN-γ: 0 36 72 0 36 72 (h)
p-MLKL
MLKL
cl. Casp-3
RIPK1
β-Actin
m
GD: – + +
MG132: – – +
RIPK3
β-Actin

Supplementary Fig. 4 Only glucose deprivation (GD) can induce necroptosis in primary mouse tumor cells. a, Primary MVT-1 tumor cells were isolated from 5-week tumors of FVB/NJ mice implanted with MVT-1 cells and treated for the indicated time points with 0.5 mM glucose (GD) and examined by Western blotting analysis of the cell lysates using the indicated antibodies. Western blotting analysis representative of three independent experiments. b, Primary MMTV-PyMT tumor cells were isolated from 15-week mice and treated for 36 hr with 0.5 mM glucose (GD) or 0.1 mM glutamine (GlnD) or 0.1% O_2 (hypoxia) and cell death was determined by PI staining using flow cytometry (left panel, n=4 biologically independent samples, each) or examined by western blotting analysis using the indicated antibodies (right panel). Data are presented as mean values +/- SEM. c, Western blotting analysis from three independent experiments of primary MMTV-PyMT tumor cells from 15-week mice under GD condition for the indicated time points using the indicated antibodies. d, Western blotting analysis from three independent experiments of primary MVT-1 cells from 5-week tumors of mice implanted with MVT-1 cells treated with GlnD (left panel) or hypoxia (right panel) for indicated time points using the indicated antibodies. e, Western blotting analysis from three independent experiments of primary MMTV-PyMT cells from 15-week mice treated with GlnD (left panel) or hypoxia (right panel) for indicated time points using the indicated antibodies. f, Western blotting analysis from three independent experiments of primary MVT-1 cells from 5-week mice implanted with MVT-1 cells treated with IFN-β (left panel) or IFN-γ (right panel) for indicated time points using the indicated antibodies. g, Western blotting analysis of primary MMTV-PyMT cells from 15-week mice treated with IFN-β (left panel) or IFN-γ (right panel) for indicated time points using the indicated antibodies. h, Western blotting analysis from three independent experiments of MVT-1 cells treated with 5-AD for 3 days, followed by GD condition for the indicated time points using the indicated antibodies. i, MVT-1 cells were transfected with shRNA control or a vector containing shRNA targeting ZBP1 sequences. For rescue cells, MVT-1 shZBP1 cells were transfected with HA-ZBP1 vector. The cells were treated with 5-AD for 3 days, followed by GD condition for 24 hr followed by Western blotting analysis using the indicated antibodies. j, Western blotting analysis from three independent experiments of B16 cells treated with 5-AD for 4 days, followed by GD condition for 24 hr was detected using the indicated antibodies. k, Primary MVT-1 CRISPR CT or ZBP1 KO tumor cells were isolated from 5-week tumors of FVB/NJ mice implanted with MVT-1 CRISPR CT or ZBP1 KO cells and under GD condition for 24 hr followed by Western blotting analysis from three independent experiments of the cell lysates using the indicated antibodies. l, Western blotting analysis from three independent experiments of MVT-1 CRISPR CT or MVT-1 RIPK1 KO cells treated with IFN-γ for 36 or 72 hr using the indicated antibodies. m, Western blotting analysis of MVT-1 cells treated with GD with or without MG132 (2 μM) until 24 hr was determined by using the indicated antibodies. Two-sided student’s t-test was used to determine the statistical significance of differences between groups. Differences with P values < 0.05 were considered significant. Source data are provided as a Source Data file.

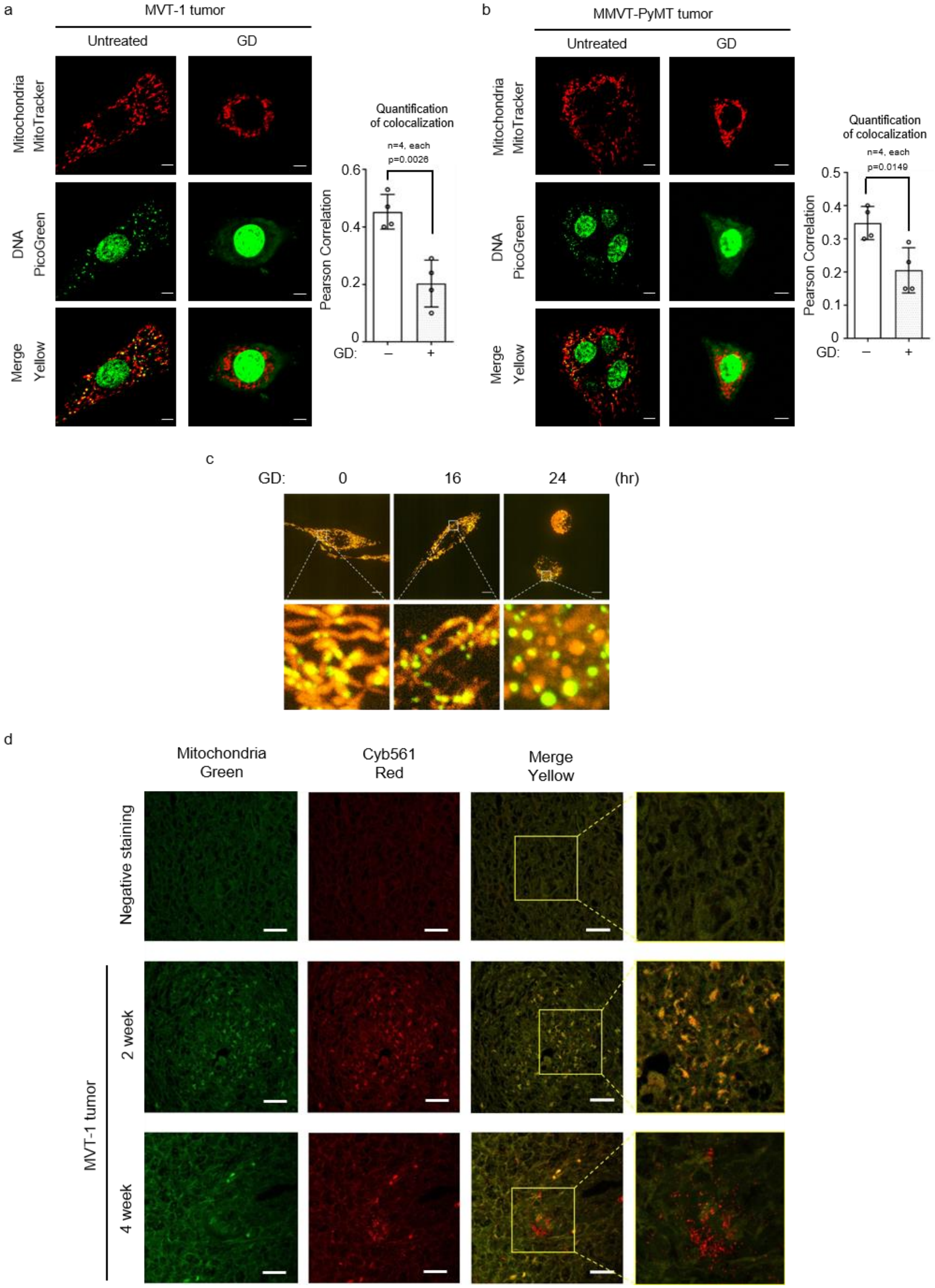
a
MVT-1 tumor
Untreated
GD
Mitochondria MitoTracker
DNA PicoGreen
Merge Yellow
Quantification of colocalization
n=4, each
p=0.0026
Pearson Correlation
GD:
b
MMVT-PyMT tumor
Untreated
GD
Mitochondria MitoTracker
DNA PicoGreen
Merge Yellow
Quantification of colocalization
n=4, each
p=0.0149
Pearson Correlation
GD:
c
GD: 0 16 24 (hr)
d
Mitochondria Green
Cyb561 Red
Merge Yellow
Negative staining
MVT-1 tumor
2 week
4 week

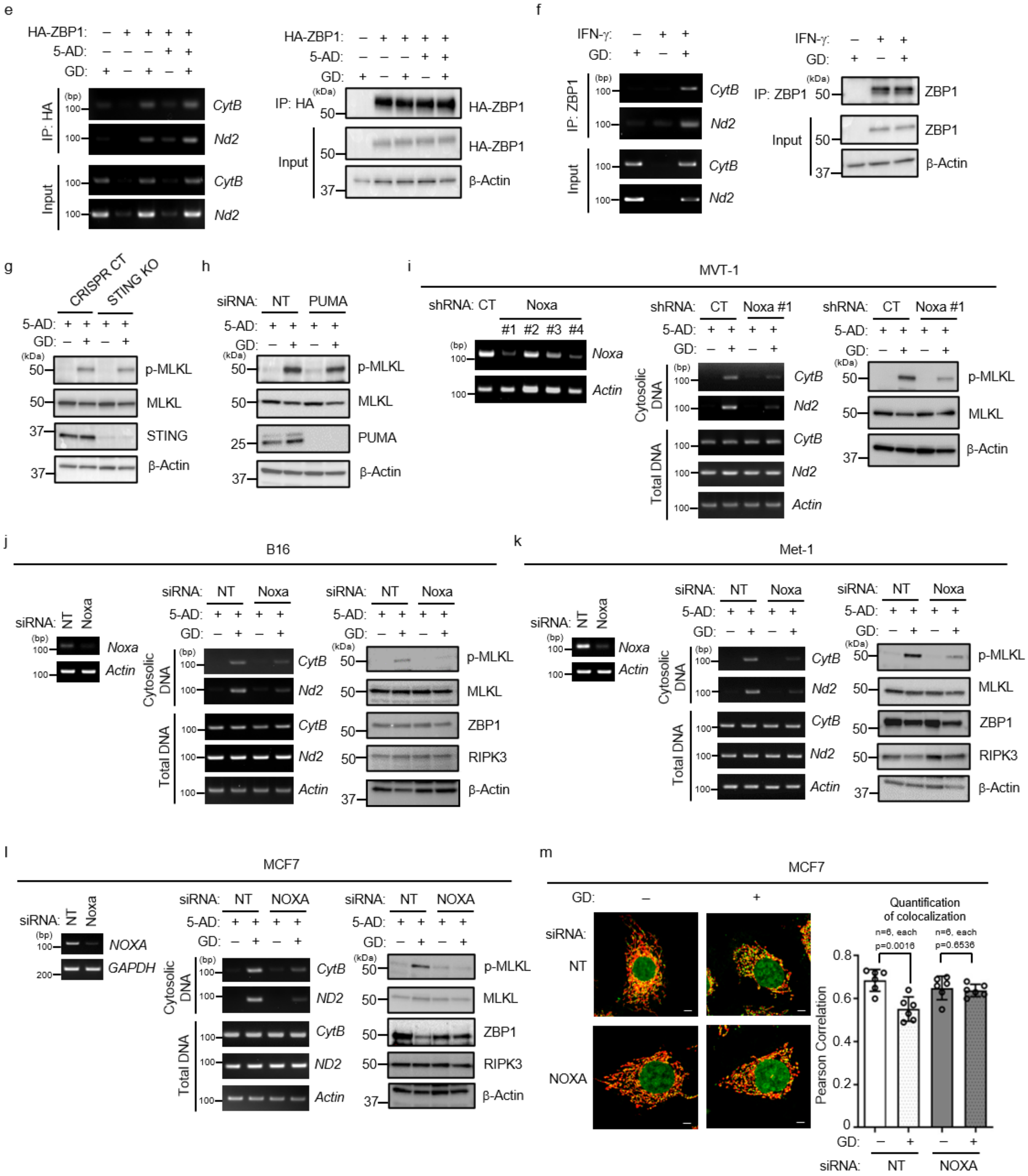

e
HA-ZBP1:
5-AD:
GD:
IP: HA
Input
CytB
Nd2
HA-ZBP1
β-Actin
f
IFN-γ:
IP: ZBP1
ZBP1
g
CRISPR CT
STING KO
p-MLKL
MLKL
STING
h
siRNA: NT PUMA
PUMA
i
MVT-1
shRNA: CT Noxa
#1 #2 #3 #4
Noxa
Actin
Noxa #1
Cytosolic DNA
Total DNA
j
B16
siRNA: NT Noxa
ZBP1
RIPK3
k
Met-1
l
MCF7
NOXA
GAPDH
ND2
m
Quantification of colocalization
n=6, each p=0.0016
n=6, each p=0.6536
Pearson Correlation

Supplementary Fig. 5 Glucose deprivation (GD) promotes mitochondrial DNA release in primary mouse tumor cells. a, Primary MVT-1 tumor cells were isolated from 5-week tumors of FVB/NJ mice implanted with MVT-1 cells and treated with 0.5 mM glucose (GD) for 16 hr. Cells were stained with MitoTracker (red) and DNA staining with PicoGreen (green) and analyzed by confocal microscopy (left panel). Scale bar, 5 µm. Quantification of colocalization of MitoTracker and PicoGreen staining was performed using Image J software (right panel, n=4 biologically independent samples, each). Data are presented as mean values +/- SEM. b, Primary MMTV-PyMT tumor cells were isolated at 15 week mice and treated with GD for 16 hr. Cells were stained with MitoTracker (red) and DNA staining with PicoGreen (green) and analyzed by confocal microscopy (left panel). Quantification of colocalization of MitoTracker and PicoGreen staining was performed using Image J software (right panel, n=4 biologically independent samples, each). Data are presented as mean values +/- SEM. c, MVT-1 cells were co-transfected with vectors targeting mtDNA (peGFP-TFAM, green) or mitochondria (pLV-mitoDsRed, red). 48 hours after the transfection, the cells were under GD condition for the indicated time. Super-resolution images were collected using the 60x objective and the SoRa spinning disk. Z-stacks were collected using a 0.2 µm step size. The images were deconvolved using a Richardson-Lucy constrained iterative algorithm included in the Nikon Elements software. Scale bar, 50 µm. d, MVT-1 tumor paraffin sections of 2-week or 4-week from mice implanted with MVT-1 cells were co-stained with mitochondria (IraZolve-Mito, green) and Cyb561 (RNA probe conjugated with Alexa Fluor 546, red) followed by manufacturer's procedure (ViewRNA ISH Tissue assay). Scale bar, 20 µm. e, MVT-1 cells stably overexpressing HA-tagged ZBP1 were treated with or without 5-Aza-2′-deoxycytidine (5-AD), and GD conditions as indicated. HA-ZBP1 was pulled down by IP with anit-HA antibody from the cytosolic fraction, followed by PCR for detecting CytB and Nd2 (left panel). Right panel shows the pull-down efficiency of anti-HA IP as a control for left panel under treatment. f, MVT-1 cells were under GD condition and/or IFN-γ. Endogenous ZBP1 was pulled down by IP with anit-ZBP1 antibody (Santa Cruz, sc-271483) from the cytosolic fraction, followed by PCR for detecting CytB and Nd2 (left panel). Right panel shows the pull-down efficiency of IP as a control for left panel under treatment. g, MVT-1 CRISPR/Cas9 control (CRISPR CT) or STING knock out (STING KO) cells were treated with 5-AD for 3 days, then under GD condition for 24 hr followed by Western blotting analysis using the indicated antibodies. h, MVT-1 cells were transfected with non-targeting siRNA (NT) or small interfering RNA targeting PUMA (siPUMA) and then treated as in g followed by Western blotting analysis using the indicated antibodies. i, MVT-1 cells were transfected with shRNA control or a vector containing shRNA targeting four different sequences of Noxa. The cells were treated as in g, then analyzed by PCR for the knockdown efficiency of Noxa (left panel) and followed by Western blotting analysis using the indicated antibodies (middle panel). Right panel shows Western blotting analysis using the indicated antibodies j, B16 cells were transfected with NT or small interfering RNA targeting Noxa (siNoxa) and analyzed by PCR for the knockdown efficiency of Noxa (left panel). The cells were further treated with 5-AD for 3 days, then under GD condition for 16 hr. Cytosolic fractions isolated from an equal number of B16 cells were

analyzed by PCR for mitochondrial CytB and Nd2 (middle panel). The cells were followed by Western blotting analysis using the indicated antibodies (right panel). k, Met-1 cells were transfected with NT or siNoxa and analyzed by PCR for the knockdown efficiency of Noxa (left panel). The cells were further treated with 5-AD for 3 days, then under GD condition for 16 hr. Cytosolic fractions isolated from an equal number of Met-1 cells were analyzed by PCR for mitochondrial CytB and Nd2 (middle panel). The cells were followed by Western blotting analysis using the indicated antibodies (right panel). l, MCF7 cells were transfected with NT or siNOXA and analyzed by PCR for the knockdown efficiency of NOXA (left panel). The cells were further treated with 5-AD for 5 days, then under GD condition for 16 hr. Cytosolic fractions isolated from an equal number of MCF7 cells were analyzed by PCR for mitochondrial CytB and ND2 (middle panel). The cells were followed by Western blotting analysis using the indicated antibodies (right panel). MCF7 cells were treated with IFN-β for 2 days to enhance ZBP1 expression. c-l, The data results representative of three independent experiments. m, MCF7 cells were transfected with NT or siNOXA and then treated with GD for 16 hr. The cells were stained with MitoTracker (red) and DNA staining with PicoGreen (green), analyzed (left panel) and quantified (right panel, n=6 biologically independent samples, each) as in a. Scale bar, 5 µm. Data are presented as mean values +/- SEM. Two-sided student's t-test was used to determine the statistical significance of differences between groups. Differences with P values < 0.05 were considered significant. Source data are provided as a Source Data file.

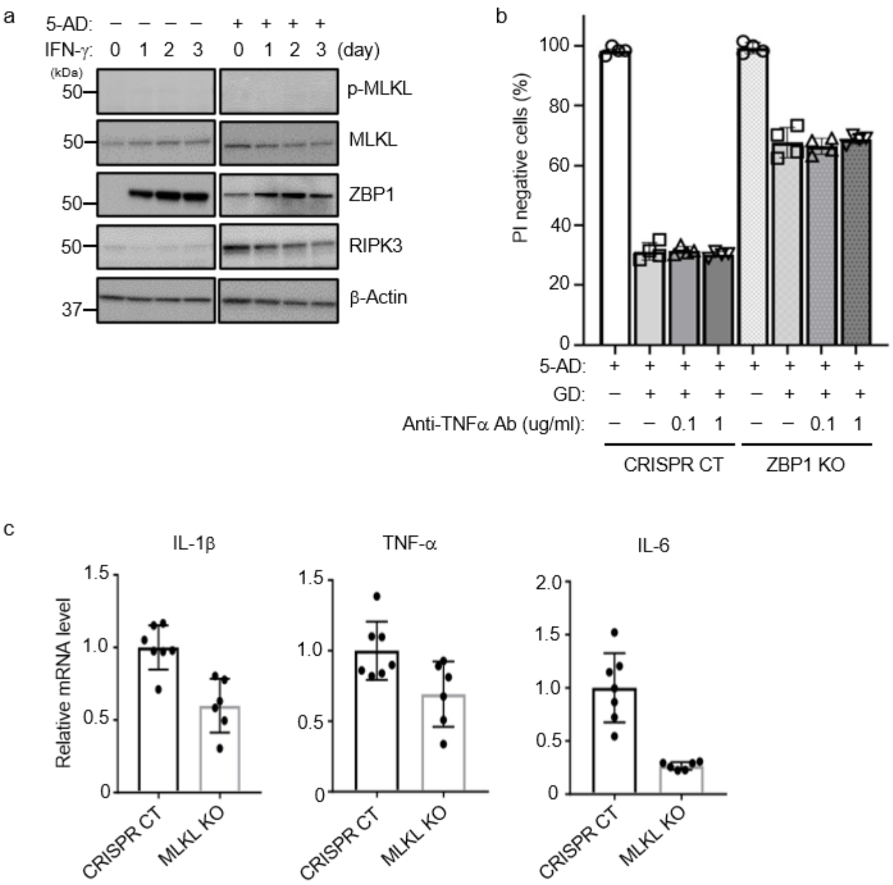
a
5-AD: – – – – + + + +
IFN-γ: 0 1 2 3 0 1 2 3 (day)
(kDa)
50
50
50
50
37
p-MLKL
MLKL
ZBP1
RIPK3
β-Actin
b
PI negative cells (%)
100
80
60
40
20
0
5-AD:
GD:
Anti-TNFα Ab (ug/ml):
0.1
1
CRISPR CT
ZBP1 KO
c
IL-1β
TNF-α
IL-6
Relative mRNA level
1.5
1.0
0.5
0
2.0
CRISPR CT
MLKL KO

Supplementary Fig. 6 Analysis of inflammatory cytokines in MVT-1 tumor model a, MVT-1 cells were treated with or without 5-Aza-2′-deoxycytidine (5-AD), for 3 days, then further treated with INF-γ for indicated time points. Western blotting analysis of the cells was detected using the indicated antibodies. Western blotting analysis is representative of three independent experiments. b, MVT-1 CRISPR CT or MVT-1 ZBP1 KO cells treated with 5-AD for 3 days, followed by GD condition for 30 hr with or without neutralized anti-TNFα antibody. Cell death analysis was determined by PI staining using flow cytometry (n=4 biologically independent samples, each). Data are presented as mean values +/- SEM. c, Quantitative real-time PCR analysis of the relative expression of IL-1β or TNF-α or IL-6 mRNA from macrophages isolated from the mice implanted with MVT-1 CRISPR CT or MLKL KO cells at 5 weeks (CRISPR CR, n=7; MLKL KO, n=6; biologically independent samples). Data are presented as mean values +/- SEM. Two-sided student's t-test was used to determine the statistical significance of differences between groups. Differences with P values < 0.05 were considered significant. Source data are provided as a Source Data file.

Supplementary Table 1

Guide RNA sequences for CRISPR knock out (5' – 3')			
	Zbp1	CACCG CAGGTGTTGAGCGATGACGG	
	Zbp1_pool2	CACCG ACCTCTTCCTTCACC TCGCG	
	Ripk1	CACCG CCAACTCACTCAGCGCGGTT	
qPCR primers (5' – 3')			
	Ripk1	Forward	GGTCAAATTCAGAACAACCTGGA
		Reverse	CACACTGCGATCATTCTCGT
	Ripk3	Forward	TCTGTCAAGTTATGGCCTACTGG
		Reverse	GGAACACGACTCCGAACCC
Methylation primers (5' – 3')			
	Ripk3	Forward	TAATTCGGAAAAAGGGTAATAATTC
		Reverse	ATAAATATCGAAAACTACGATCGAC
Unmethylation primers (5' – 3')			
	Ripk3	Forward	AATTTGGAAAAAGGGTAATAATTTG
		Reverse	ATAAATATCAAAAACTACAATCAAC
Primers for basic PCR (5' – 3')			
	Trif	Forward	AACCTCCACATCCCCTGTTTT
		Reverse	GCCCTGGCATGGATAACCA
	Actin	Forward	CTGCCTGACGGCCAGG
		Reverse	CTATGGCCTCAGGAGTTTTGTC
	hGAPDH	Forward	GTATTCCCCCAGGTTTACAT
		Reverse	TTCTGTCTTCCACTCACTCC
	Noxa	Forward	GCAGAGCTACCACCTGAGTTC
		Reverse	CTTTTGCGACTTCCCAGGCA
	hNOXA	Forward	CTGGAAGTCGAGTGTGCTACTC
		Reverse	TGAAGGAGTCCCCTCATGCAAG
	CytB	Forward	CCACTTCATCTTACCATTTATTATCGC
		Reverse	TTTTATCTGCATCTGAGTTTAA
	Nd2	Forward	CCTGTAATCACAATATCCA
		Reverse	CTGTTGCTTGTGTGACGAAG
	hCytB	Forward	GCCTGCCTGATCCTCCAAAT
		Reverse	AAGGTAGCGGATGATTCAGCC
	hND2	Forward	CCCTAAAACCCGCCACATCT
		Reverse	AGAGCGATGGTGAGAGCTAAGG
