# Supplementary material for: ZBP1 not RIPK1 mediates tumor necroptosis in breast cancer
Source: Nat Commun. 2021 May 11;12:2666. doi: 10.1038/s41467-021-23004-3 (PMC8113527; doi:10.1038/s41467-021-23004-3)
Supplement: Supplementary file 3 — Reporting Summary [file 41467_2021_23004_MOESM3_ESM.pdf]

nature research

Corresponding author(s): DBPR, NCOMMS-20-13376D

Last updated by author(s): Mar 22, 2021

Reporting Summary

Nature Research wishes to improve the reproducibility of the work that we publish. This form provides structure for consistency and transparency in reporting. For further information on Nature Research policies, see Authors & Referees and the Editorial Policy Checklist.

Statistics

For all statistical analyses, confirm that the following items are present in the figure legend, table legend, main text, or Methods section.

n/a	Confirmed	
☐	☒	The exact sample size (*n*) for each experimental group/condition, given as a discrete number and unit of measurement
☐	☒	A statement on whether measurements were taken from distinct samples or whether the same sample was measured repeatedly
☐	☒	The statistical test(s) used AND whether they are one- or two-sided<br>*Only common tests should be described solely by name; describe more complex techniques in the Methods section.*
☒	☐	A description of all covariates tested
☒	☐	A description of any assumptions or corrections, such as tests of normality and adjustment for multiple comparisons
☐	☒	A full description of the statistical parameters including central tendency (e.g. means) or other basic estimates (e.g. regression coefficient) AND variation (e.g. standard deviation) or associated estimates of uncertainty (e.g. confidence intervals)
☐	☒	For null hypothesis testing, the test statistic (e.g. *F*, *t*, *r*) with confidence intervals, effect sizes, degrees of freedom and *P* value noted<br>*Give P values as exact values whenever suitable.*
☒	☐	For Bayesian analysis, information on the choice of priors and Markov chain Monte Carlo settings
☒	☐	For hierarchical and complex designs, identification of the appropriate level for tests and full reporting of outcomes
☐	☒	Estimates of effect sizes (e.g. Cohen's *d*, Pearson's *r*), indicating how they were calculated

Our web collection on statistics for biologists contains articles on many of the points above.

Software and code

Policy information about availability of computer code

Data collection	qPCR were performed using SensiFAST Probe Hi-ROX Mix (Bioline) on QuantStudio 3 Real-Time PCR System (Thermo Fisher Scientific). .<br>We included a statement in the Methods/ RNA Isolation and Quantitative PCR (qPCR).<br>PCR to identify methylated versus unmethylated RIPK3 - primer sequences are included in the methods section.<br>Immunofluorescence images were acquired using a ZEISS LSM 880 (Zeiss) confocal microscope.<br>Super-resolution microscopy images were acquired using a Nikon Ti-2 microscope equipped with a Yokogawa CSU-W1 SoRa spinning disk unit, 20x plan-apochromat (N.A. 0.75) and 60x plan-apochromat (N.A. 1.49) objective lenses, and Photometrics BSI sCMOS camera.<br>RNA-Seq data from The Cancer Genome Atlas (TCGA) Research Network was retrieved from NCI’s Genomic Data Commons (GDC) repository using the TCGABiolinks R package .
Data analysis	The statistical analysis was performed using GraphPad Prism (Version 8). We included a statement in the Methods/Statistical Analysis.<br>Colocalization of MitoTracker Red and PicoGreen was quantified by analysis of Pearson’s correlation using Image J software<br>For RNA-Seq Analysis of TCGA Data we used P-values that were adjusted for using the Benjamini-Hochberg procedure.<br>The quantification of tumor necrotic/death area was counted using Image J and represented as the percentage of tumor necrotic/death area within whole tumor.<br>Super-resolution microscopy images were deconvolved using a Richardson-Lucy constrained iterative algorithm included in the Nikon Elements software.

For manuscripts utilizing custom algorithms or software that are central to the research but not yet described in published literature, software must be made available to editors/reviewers. We strongly encourage code deposition in a community repository (e.g. GitHub). See the Nature Research guidelines for submitting code & software for further information.

Data

Policy information about availability of data

All manuscripts must include a data availability statement. This statement should provide the following information, where applicable:

- Accession codes, unique identifiers, or web links for publicly available datasets
- A list of figures that have associated raw data
- A description of any restrictions on data availability

Primer sequences, antibodies and CRISPR guide RNA sequences used in this study are provided in the Methods section. The data that support the findings of this study are available from the corresponding author upon request.

Field-specific reporting

Please select the one below that is the best fit for your research. If you are not sure, read the appropriate sections before making your selection.

☒ Life sciences ☐ Behavioural & social sciences ☐ Ecological, evolutionary & environmental sciences

For a reference copy of the document with all sections, see nature.com/documents/nr-reporting-summary-flat.pdf

Life sciences study design

All studies must disclose on these points even when the disclosure is negative.

Sample size	We chose the sample size of animal experiments according to similar studies in the field. We usually use at least 5 mice per group. For cell and biochemical experiments, our data represent at least three independent assays. The number of the independent experiments was indicated in each figure legend.
Data exclusions	No data were excluded from analysis.
Replication	Experimental data was reliably reproduced in multiple independent experiments. For all experiments, our data represent at least three independent assays that produce similar results. We also used different assays and readouts to confirm our findings in different ways. The number of the independent experiments was indicated in each figure legend.
Randomization	Mice were allocated into groups according to genotype of interest and were allocated into groups randomly.
Blinding	Investigators were blinded during the data collection and analysis where possible. This included mice data collection, histological analysis etc. The quantification for immunofluorescence assays was performed blindly.

Reporting for specific materials, systems and methods

We require information from authors about some types of materials, experimental systems and methods used in many studies. Here, indicate whether each material, system or method listed is relevant to your study. If you are not sure if a list item applies to your research, read the appropriate section before selecting a response.

Materials & experimental systems

n/a	Involved in the study	
☐	☒	Antibodies
☐	☒	Eukaryotic cell lines
☒	☐	Palaeontology
☐	☒	Animals and other organisms
☒	☐	Human research participants
☒	☐	Clinical data

Methods

n/a	Involved in the study	
☒	☐	ChIP-seq
☐	☒	Flow cytometry
☒	☐	MRI-based neuroimaging

Antibodies

Antibodies used	Anti-ZBP1 (AG-20B-0010) for mouse from AdipoGen; p-MLKL (ab196436) for mouse and MLKL (184718) from Abcam; RIPK3 (2283) for mouse from ProSci; RIPK1 (610459) from BD Biosciences; cl.Casp-3 (9664) and Casp-3 (14220) and HIF1-[illegible] (3434) from Cell Signaling Technology; HA (sc-80s) from Santa Cruz Biotechnology; Anti-actin (A3853) from Sigma. Anti- ZBP1 (SC-271483) for mouse from Santa Cruz Biotechnology<br>Anti-ZBP1 (AF6309) for human from R&D systems; anti-RIPK3 (ab72106) for human from Abcam; Ki-67 (12202S), anti-STING (13647), and anti-PUMA (14570S) from Cell Signaling Technology. anti-DNMT1 (5032) for mouse and human from Cell Signaling Technology
Validation	Validation for all commercial antibodies used in this study can be found in manuacturer's website.

Eukaryotic cell lines

Policy information about cell lines

Cell line source(s)	MVT-1, B16, LLC, MCF7, MDA-MB-231 were purchased from ATCC. We included a statement in the Methods. Met-1 cell line was a gift from K. Kelly, NCI, NIH.
Authentication	All cell lines have been purchased from ATCC and authenticated by this organization with certificates.
Mycoplasma contamination	All cells tested negative for mycoplasma.
Commonly misidentified lines (See ICLAC register)	No commonly misidentified cell lines were used in this study.

Animals and other organisms

Policy information about studies involving animals; ARRIVE guidelines recommended for reporting animal research

Laboratory animals	Mice Female FVB/NJ, and FVB/N-Tg (MMTV-PyVT) 634Mul/J (MMTV-PyMT) and C57BL/6J and BALB/c-nu/nu mice were purchased from The Jackson Laboratory. All animal experiments were performed under protocols approved by National Cancer Institute Animal Care and Use Committee and followed NIH guidelines. Only female mice of 6-8 weeks for each strain were used for experiments except for FVB/N-Tg (MMTV-PyVT) 634Mul/J (MMTV-PyMT) female mice which were collected at 10- 15 weeks of age. The age of the mice at the time of experiment is indicated in the Methods.
Wild animals	The study did not involve wild animals.
Field-collected samples	The study did not involve samples collected from the field.
Ethics oversight	All animal experiments were performed under protocols approved by National Cancer Institute Animal Care and Use Committee and followed NIH guidelines.

Note that full information on the approval of the study protocol must also be provided in the manuscript.

Flow Cytometry

Plots

Confirm that:

☒ The axis labels state the marker and fluorochrome used (e.g. CD4-FITC).

☒ The axis scales are clearly visible. Include numbers along axes only for bottom left plot of group (a 'group' is an analysis of identical markers).

☐ All plots are contour plots with outliers or pseudocolor plots.

☒ A numerical value for number of cells or percentage (with statistics) is provided.

Methodology

Sample preparation	MVT-1 cells were lentiviral transfected with CMV-GFP-T2A-Luciferase Lentivector (SBI system biosciences), successfully transfected MVT-1 cells were GFP positively sorted by using BD FACSAria Fusion sorter (BD bioscience). Then, GFP-MVT-1 cells (2×106) were injected into fat pad of FVB/NJ mice. After 5 weeks, tumor was collected and digested in PBS buffer as in above described. The GFP positive MVT-1 cells were then sorted from total cells by using a BD FACSAria Fusion sorter.<br>Proliferation and cell cycle of MVT-1 CRISPR CT or MVT-1 RIPK1 KO cells were analyzed by BrdU-FITC uptake and 7-AAD-APC staining using flow cytometry.<br>Primary MVT-1 tumor cells were isolated at 5 weeks post-implantation and treated for 36 hr with 0.5 mM glucose (GD) or 0.1 mM glutamine (GlnD) or 0.1 % O2 (hypoxia) and cell death was determined by PI staining using flow cytometry .
Instrument	For cell sorting we used BD FACSAria Fusion sorter (BD bioscience). Flow cytometry analysis was performed on Sony SA3800.
Software	All flow cytometry analysis was done using BD CellQuest Pro and FlowJo.
Cell population abundance	The relevant post-sort date and how it was performed is described in detail in Supplementary Figure 2 C and D
Gating strategy	The relevant post-sort date and how it was performed is described in detail in Supplementary Figure 2 C and D

☒ Tick this box to confirm that a figure exemplifying the gating strategy is provided in the Supplementary Information.
